# Choice behavior in autistic adults: What drives the extreme switching phenomenon?

**DOI:** 10.1371/journal.pone.0282296

**Published:** 2023-03-02

**Authors:** Dana Zeif, Ofir Yakobi, Eldad Yechiam

**Affiliations:** 1 Max Wertheimer Minerva Center for Cognitive Studies, Technion – Israel Institute of Technology, Haifa, Israel; 2 Department of Psychology, Haifa University, Haifa, Israel; Children’s Hospital of Los Angeles, UNITED STATES

## Abstract

**Background:**

Previous studies reported that autistic adolescents and adults tend to exhibit extensive choice switching in repeated experiential tasks. However, a recent meta-analysis showed that this switching effect was non-significant across studies. Furthermore, the relevant psychological mechanisms remain unclear. We examined the robustness of the extreme choice-switching phenomenon, and whether it is driven by a learning impairment, feedback-related aspects (e.g., avoiding losses), or alternatively a different information sampling strategy.

**Methods:**

We recruited an online sample of 114 US participants (57 autistic adults and 57 non-autistic). All participants performed the Iowa Gambling task, a four-option repeated choice task. Standard task blocks were followed by a trial block with no feedback.

**Results:**

The findings replicate the extreme choice switching phenomenon (Cohen’s *d* = 0.48). Furthermore, the effect was found with no difference in average choice rates denoting no learning impairment, and was even observed in trial blocks with no feedback (*d* = 0.52). There was no evidence that the switching strategy of autistic individuals was more perseverative (i.e., that similar switching rates were used in subsequent trial blocks). When adding the current dataset to the meta-analysis, the choice switching phenomenon is significant across studies, *d* = 0.32.

**Conclusions:**

The findings suggest that the increased choice switching phenomenon in autism may be robust and that it represents a distinct information sampling strategy and not poor implicit learning (or a bias in the sensitivity to losses). Such extended sampling may underlie some of the phenomena previously attributed to poor learning.

## Background

Autism, also referred to as Autism Spectrum Disorder, is a heterogeneous condition characterized by restricted interests and behaviors, as well as deficits in social communication. It affects approximately one in 44 children in the United States [1]. Paradoxically, despite the tendency towards restricted and repetitive behavior found in clinical studies [2, 3], some studies of decision making have uncovered an elevated tendency to shift between different choice options in repeated choices [4, 5], namely to cover more options by switching between them. Initially, Johnson et al. [4] examined the behavior of autistic adolescents and young adults with no language delay in the Iowa Gambling Task (IGT; [6]), a repeated choice task involving four options where two of the options are advantageous and two are disadvantageous (see details in Table 1). Autistic adolescents were found to have no significant difference in the rate of advantageous choices compared to those with no autism, but at the same time switched choices (i.e., selected a different option in trial *t* than the one chosen in trial *t*—1) in 84% of their choices, while non-autistic adolescents switched in 51% of their choices. The increased switching phenomena in autism has been found in other tasks and domains as well, such as two-option tasks [5], spatial rout selection [7], and the selection of social agents [8]. Yet the question remains whether this is simply the result of noise in the selection process (e.g., due to poorer learning) or actually a more extensive sampling strategy. The goal of the current study was to examine the robustness of this phenomenon given several null findings (elaborated next), and shed light on its psychological mechanisms.

**Table 1:** The payoffs in the Iowa Gambling Task (IGT).

Deck	Outcome	Description
**A**	Win $100 every card and .5 probability to lose $250	Disadvantageous
**B**	Win $100 every card and .1 probability to lose $1,250	Disadvantageous
**C**	Win $50 every card and .5 probability to lose $50	Advantageous
**D**	Win $50 every card and .1 probability to lose $250	Advantageous

The difference in choice switching on the IGT between autistic and non-autistic individuals has been replicated in some studies [9, 10], though not in others [11, 12]. A recent meta-analysis [13] concluded that the main effect across studies was not significant, but only six studies with switching data were identified and their results were highly heterogeneous, with three showing a significant effect and three that did not. The studies were also different in their inclusion criteria, for example some studies excluded individuals with autism and OCD whose checking behaviors might conceivably result in higher switching [14]. Moreover, four of the studies had small sample sizes in the autism group (n < 25), stressing the need for additional empirical investigation.

In addition, as noted above the cognitive and emotional underpinnings of the increased choice switching phenomenon in autism are yet unclear. Several lines of explanations have been proposed. First, it has been suggested that increased switching is a by-product of a difficulty to implicitly learn to select the best option when options provide fuzzy and inconsistent outcomes [4, 10]. To put it simply, implicit learning is learning that takes place outside of conscious awareness [15]. It has been previously argued that some of the social and behavioral difficulties in autism are due to an impairment in this capacity owing to basal ganglia abnormalities (see review in [16]). This notion was supported by Mussey et al. [10] in showing that young autistic adults performed worse than a non-autism group on the IGT, and importantly, that for autistic individuals elevated switching was correlated with fewer advantageous choices at the end of the learning period (i.e., in the final block of trials). However, in Johnson et al. [4] switches were not correlated with significantly poorer (or indeed higher) outcomes on a given trial (the mean correlation between switching and outcome size on a given trial was 0.01 in the autism group and -0.01 in the non-autism group).

Alternatively, it has been suggested that the elevated switching may be driven by sensitivity to some aspects of feedback, especially negative outcomes [4, 17]. This suggests that the only reason that autistic participants switch in the IGT is because choice options in this task (even advantageous ones) produce occasional losses, whereas in a clinical setting individuals might stick to options where there is no loss, which would be consistent with the observed tendency of autistic adults to avoid changing routines when possible [18]. Using a reinforcement learning model, Johnson et al. [4] found that the weight of loss outcomes was larger for autistic adolescents than for age-matched peers. However, Johnson et al. [4] interestingly did not find that switching responses of autistic individuals occurred more strongly following losses than gains (and see also [10]).

A third and different explanation is that autistic individuals have a different strategy of sampling information, either due to elevated curiosity [9, 19] or a rigid and inflexible strategy of checking different options during learning [10]. This account implies that the elevated switching effect is not a secondary phenomenon driven by a learning impairment, but rather a separate phenomenon having to do with extensive sampling of options (i.e., exploration) which leads to poor or better performance depending on the situation. Considerable sampling should in theory improve decision performance when limited exploration produces inefficient solutions [20, 21]. For example, self-identified autistic users were found to look at more Internet search results for a given query [22]; this strategy is adaptive as long as searching the initial search entries is inefficient. As noted, elevated sampling could be the result of sheer curiosity or a rigid and compulsive strategy. Rigidity in a sampling strategy, namely perseveration and routine/repetition, would imply that the same or similar *strategy* of switching options is continually used by an individual (be it low or high sampling). This should be reflected in more stable switching rates across subsequent blocks in the autism group.

In the current study we examined the robustness of the extreme choice-switching phenomenon in an online sample of previously diagnosed participants. We also examined whether this behavioral tendency emerges concurrently (or not) with reduced advantageous choices and reflects impaired learning. In addition, we examined whether the elevated switching pattern is driven by aspects of the choice feedback, such as losses, by testing the replicability of Johnson et al.’s [4] modeling results and also by supplementing the IGT with a trial block with no feedback and examining whether the extensive switching phenomenon persists in this block as well (see related manipulations in [23, 24]). Finally, we also evaluated the perseverance of switching in different trial blocks and its association with OCD.

## Methods

### Participants

The study was preapproved by the Technion Medical Research Ethics Committee. It was pre-registered at clinicaltrials.gov (NCT04631432). Participants responded to a daily ad in Amazon Mechanical Turk (MTurk) regarding a paid experiment in decision making. The version of the ad for the autism group also included the inclusion criterion of having a prior diagnosis of autism and a request to upload a preexisting diagnosis certificate (which was anonymized by deleting the participant’s name). All uploaded diagnoses were reviewed, and individuals who submitted documents that were not a diagnosis were excluded. All certificates were signed by psychiatrists or psychologists, or were issued by the state. No minors were allowed. We originally aimed for 200 autistic participants (based on the limitation of our resources [25]) but as it turned out, the number of autistic individuals in MTurk who agreed to participate was smaller, and we therefore stopped running the study at the end of the IRB deadline. The number of participants in the autism group was 57 (29 males, 26 females, and 2 others). Given a potentially medium effect size (d = 0.5), a power of 80%, and a group-size ratio of 1:1 this produces a statistical power of 75%.

Recruitment of participants to the non-autism group took place in two waves: Three months after beginning the study, and at the end of the autism group recruitment period. Participants in the non-autism group were matched to the autism group by gender and age. Matching by gender was administered by recruiting a male/female non-autistic participant for each male/female in the autism group, respectively. Age matching was achieved by setting the minimum and maximum age of the non-autism group to that of the initial sample of autistic participants (recruited during three month). The age limits we imposed were 18 to 47 years old. The number of participants in the non-autism group was identical to their number in the autism group (57 in total, 30 males and 27 females). In order to increase homogeneity, participation was restricted to US citizens. Participants received $8 for taking part in the experiment plus an additional amount of 0 to $5 reflecting their earning in the IGT.

### Experimental tasks

All tasks were delivered online. Participants first signed a written consent form and completed demographic information (e.g., age, education, gender, prior diagnoses). Those in the autism group also uploaded their diagnosis certificate (see details in S1 File). All participants then performed the IGT. In this task participants are presented with four decks of cards labeled A, B, C, and D. They can select a single card from these four decks in each trial. After selecting each card, participants receive token money (the amount is displayed on the screen). Task payoffs are presented in Table 1 and screen illustrations appear in S1 File. Two of the decks are advantageous and produce lower gains but somewhat lower (uncertain) losses; these have positive expected values. The other two decks are disadvantageous and produce higher gains but also higher (uncertain) losses; these have negative expected values. The cumulative payoff is presented at the bottom of the display and is updated at the end of each trial. The display also includes the amount given to participants at the beginning of the task as a “loan”. The initial loan in our study was $3500. The minimum inter-trial interval was set to one second, and the task included 120 trials, which were analyzed by dividing them into four 30-trials blocks. Participants were given verbal instructions identical to those provided in Johnson et al. [4] (see S1 File). Following the standard version of the IGT there was another block of trials with no payoff feedback. This trial block was administered at the end so that participants first learn the incentive structure of the task. Given the fact that there was no feedback we felt that 30 trials would be sufficient to gage the participants’ responses. Prior to this no-feedback block, participants were instructed that over the next trials they would not receive any payoff feedback. Amounts were converted to actual money at the end of the task at a rate of $1 for each $1500 of token money.

Next, participants completed verbal and non-verbal brief intellectual aptitude tests. The verbal test was the Multidimensional Aptitude Battery (MAB; [26]), a modified Similarities subscale from the Wechsler Abbreviated Scale of Intelligence (WASI; [27]). The non-verbal test was the Raven Standard Progressive Matrices (RSPM, Set 1; [28]).

Finally, in order to validate group differences, we administered additional self-report questionnaires for autism-related symptoms, the Autism Spectrum Quotient (AQ10) [29] and the Social Responsiveness Scale, 2nd Edition (SRS-2) (adult self-report version [30]). The AQ10 is a self-administered ten-item questionnaire used for measuring where adults lie on the autism spectrum or continuum. Though there are some findings questioning the reliability and validity of the AQ10 [31, 32], we used it as a brief validation of the documented diagnosis. The SRS-2 is a 65-item questionnaire that assesses difficulties in reciprocal social behavior that lead to interference with everyday social interactions.

### Analysis

Our main analysis focused on the IGT. As indicated in the pre-registration protocol, we used repeated measures analyses of variance (ANOVAs), with two dependent measures: Choice rates from the advantageous decks and choice switching rates (proportions), in each block of thirty trials. Because these two outcome measures were based on the same data, we applied Bonferroni corrections for the significance criteria in all statistical tests comparing groups on these variables (p < .025; two tailed). This analysis was also separately conducted for the no-feedback block. Additionally, we examined the correlations between choice switching and advantageous deck selection, to assess their inter-correlation. In secondary analyses, in order to examine the possibility that the sampling strategy is more rigid in autistic individuals, we also evaluated the perseverance of choice switching by examining the average change in switching rate in subsequent block of trials (absolute different between block t and block t+1). In the supplementary section (S2 File) we also include cognitive modeling analysis of the data using the Expectancy-Valence Prospect-Theory (EV-PT) model [33], which further evaluates the sensitivity to losses in the autism group. Finally, we also examined the correlation between choice switching and the two self-report questionnaires of autism-related symptoms, as well as the IQ tests and prior psychiatric diagnoses.

## Results

### Group characteristics

Table 2 presents the participants’ characteristics in the two groups. As can be seen, participants in the autism group scored higher on the AQ10 and SRS-2 while being not significantly different from the non-autism group in age, education, and general intellectual capacities. In the autism group, 75.4% of the participants scored 6 or higher in the AQ10, the cut-off previously found to be indicative of autism diagnosis [29], compared to only 10.5% in the non-autism group (*χ*^2^(1) = 49.00, *p* < .001). In the SRS-2, 87.7% of the participants in the autism group were characterized by moderate to severe interference with social interactions (i.e., a score of 85 and higher [30]). By contrast, in the non-autism group, 35.1% of the participants self-reported such difficulties (*χ*^2^(1) = 33.31, *p* < .001; see Discussion).

**Table 2:** Participants’ characteristics in the two study groups. Standard deviations appear in parentheses.

Characteristic	Non-Autism	Autism
	(n = 57)	(n = 57)
**Gender (% male)**	52.6%	50.9%
**Education (% college)**	64.9%	61.4%
**Age**	33.66 (6.39)	31.07 (7.83)
**AQ10** **	2.58 (1.89)	6.7 (2.49)
**SRS-2** **	75.45 (23.81)	110.59 (25.05)
**MAB**	23.1 (7.99)	21.87 (7.64)
**RSPM**	7.21 (2.56)	6.47 (2.74)

Notes:

* = p < .05;

** = p < .001 (difference between study groups; Bonferroni corrected).

AQ10 = Autism Spectrum Quotient, SRS-2 = Social Responsiveness Scale, 2nd Edition, MAB = Multidimensional Aptitude Battery; RSPM = Raven Standard Progressive Matrices.

Additionally, we checked for comorbid disorders. One individual from the autism group and one individual from the non-autism group reported a history of schizophrenia. In line with the study protocol, they were not excluded. Additionally, 18 individuals in the autism group (31.5%) reported also being diagnosed with obsessive compulsive disorder (OCD) compared to three in the non-autism group (5.2%). Also, 12 individuals in the autism group (21.1%) reported that they had ADHD compared to none of the non-autistic participants. This is consistent with the generally higher rate of OCD and ADHD among autistic adults [34, 35].

### Standard Iowa Gambling Task

**Fig 1:**
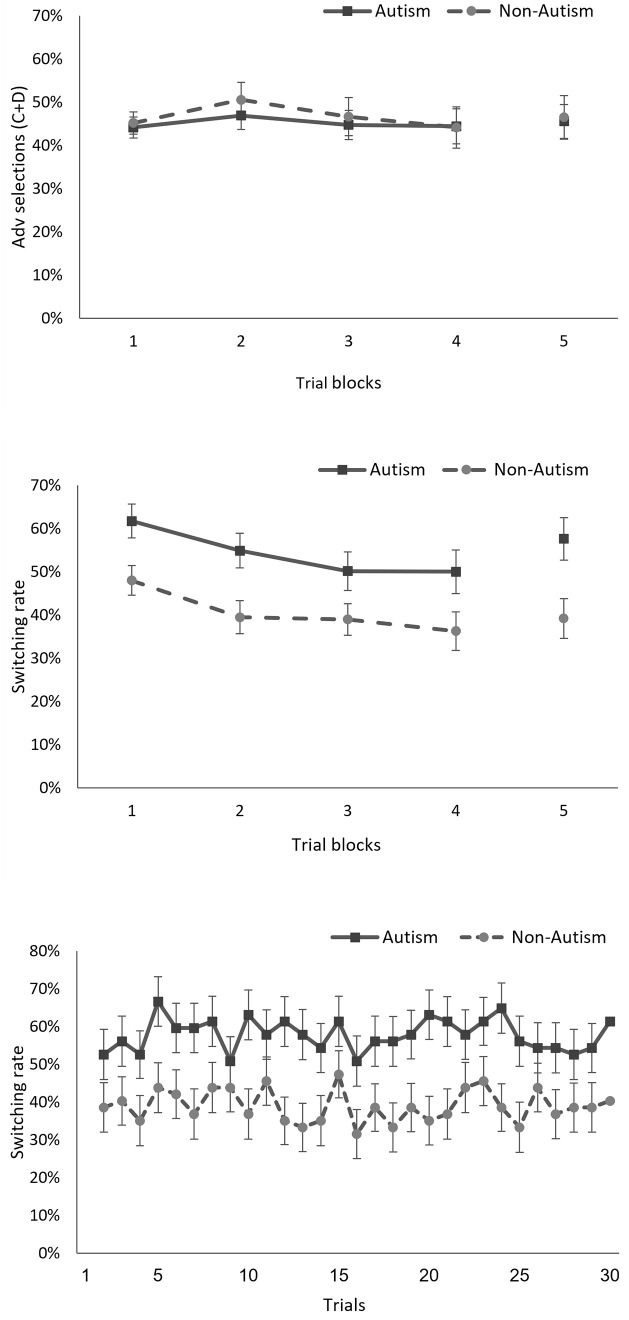
Results from the Iowa Gambling Task (IGT). Top panel: Percentages of advantageous selections in five trial blocks (four blocks of 30 trials in the standard IGT followed by a single no-feedback block). Middle panel: Switching rates in the five trial- blocks. Bottom panel: Switching rates during the 30 trials with no feedback. Error terms denote standard errors.

Fig 1 presents the results from the IGT. Though the data was slightly skewed (advantageous selections: Pearson *S*_k1_ = 0.12; choice switching: Pearson *S*_k1_ = 0.12), simulation studies have shown that ANOVA is robust to moderate skewness [36, 37], and thus we used the pre-registered ANOVA. As can be seen in the figure’s top panel, there was only a small difference between conditions in participants’ advantageous selections throughout the task. The ANOVA results for advantageous choices showed no difference between the autism and non-autism groups across trials, *F*(1,112) = 0.15, *p* = .70, and no main effect of trial block, *F*(3, 336) = 1.20, *p* = .31, as well as no interaction between study group and trial block, *F*(3,336) = 0.20, *p* = .89. We also looked at the decks producing better outcomes most of the times (B and D). The results similarly showed no difference between the two groups in mean choices from these decks, *F*(1, 112) = 0.17, *p* = .68, and no trial block by group interaction, *F*(3, 336) = 0.94, *p* = .42.

Simultaneously with the absence of a difference in rates of advantageous deck selections, the middle panel of Fig 1 shows a consistent difference between groups in choice switching. On average, participants in the autism group switched choices in 54.1% of their selections, compared to 40.6% in the non-autism group, and thus participants in the autism group made on average 1.33 more switching decisions. The ANOVA showed that this difference was statistically significant, *F*(1, 112) = 6.64, *p* = .01, with a Cohen’s *d* of 0.48, denoting a medium sized effect. Additionally, there was a main effect of trial block, *F*(3, 336) = 12.33, *p* < .001, with fewer switches as the task progressed as commonly found in repeated choice tasks (e.g., [38]). The interaction between study group and the effect of trial block on choice switching was not significant, *F*(3, 336) = 0.34, *p* = .79.

We also examined the correlation between choice switching and deck selection at the individual level. For the autism group, there was no significant correlation between switching and advantageous selection, *r* = .13, *p* = .34. For the non-autism group the correlation between these indices was also not significant, *r* = -0.12, *p* = .37. The difference between these two correlations did not reach significance, *Z* = 1.41, *p* = .16. Mussey et al. [10] found significant negative correlation in the last block of trials. However, focusing on the latter block replicates the non-significant findings (autism: *r* = -0.10, *p* = .46; non-autism: *r* = .15, *p* = .27, difference: *Z* = 1.30, *p* = .19).

Next, we examined the perseverance of the switching rate in different trial blocks. The average absolute difference between subsequent blocks in switching rates was similar in both groups (autism: 0.135, non-autism: 0.139) and the difference was non-significant, *t*(112) = 0.21, *p* = .84, providing no evidence for greater perseverance in individuals’ switching strategy in the autism group. We also examined the relative similarity in switching rates by calculating the mean correlation between switching rates in subsequent blocks. The results showed a slightly higher correlation in the autism group than in the non-autism group (*r* = 0.83, 0.76, respectively), but the difference was not significant (*Z* = 1.0, *p* = .32).

Finally, our cognitive modeling analysis (expanded in S2 File), applied to the feedback trial blocks, did not replicate the results of Johnson et al. [4]: It indicated that the autism group did not show significantly increased loss aversion, namely more sensitivity to losses than the non-autism group.

### No feedback block

As shown in Fig 1 top and middle panel, the results for the no-feedback block were similar to those of the standard IGT. Namely, on the one hand there was no significant difference between the autism and non-autism group in the rate of selections from advantageous decks, *t*(112) = 0.15, *p* = .58. There was also no difference in the rate of selecting decks with commonly rewarding outcomes (B+D), *t*(112) = 1.27, *p* = .21. On the other hand, there was significantly more choice switching in the autism group, *t*(112) = 2.75, *p* = .007. The difference between the two groups in choice switching was similar to that found in the standard-trials blocks, with a Cohen’s *d* of 0.52. Also, as indicated in Fig 1 bottom panel the difference was stable across the 30 trials with no feedback.

Also, unexpectedly, for both groups there was a slight increase in choice switching from the last block of standard trials to the block of no-feedback trials. This increase was significant, *F*(1, 158) = 8.47, *p* = .004; and was not qualified by study group, *F*(1, 158) = 1.67, *p* = .20.

### Additional findings

Next, we looked at the correlations between switching rates (in the standard IGT blocks) and other variables within the autism group. In the autism group, we found no correlation between switching and the AQ10, *r* = 0.14, *p* = .36; as well as SRS-2, *r* = 0.04, *p* = .79. S1 Fig presents the relevant scatterplots. Higher switching was also not significantly correlated the Multidimensional Aptitude Battery scores, *r* = 0.17, *p* = .26; and Raven’s matrices scores, *r* = -0.28, *p* = .07. There was also no difference in choice switching between individuals in the autism group with ADHD and those without (n = 12, 45, respectively), *t*(55) = 0.09, *p* = .93.

We did find that switching was higher for individuals in the autism group with a self-reported diagnosis of OCD, *t*(55) = 2.07, *p* = .04. Indeed, when we separately examined participants in the autism group with OCD and those without (n = 18, 39, respectively), there was a significant difference in switching rates between the autism plus OCD subsample and the non-autism group, *t*(73) = 3.77, *p* < .001, but not between the autism without OCD subsample and the non-autism group, *t*(94) = 1.36, *p* = .18. Given this finding, we conducted an exploratory ANOVA to directly examine the effect of autism and OCD as independent factors on choice switching. The results show that the effect of autism remained significant, *F*(1, 110) = 6.10, *p* = .02, replicating our main analysis, but the effect of OCD was not, *F*(1, 110) = 0.07, *p* = 0.80.

We also looked at the correlates of choice switching in the non-autism group. Interestingly, here we did find a strong positive correlation between choice switching and the SRS-2, *r* = 0.38, *p* = .01. This was not due to greater variance in the SRS-2 in the non-autism group: a Levene test for variances indicated no significant difference in this respect, *F*(112) = 0.002, *p* = .96 (see scatter plot in S1 Fig). Higher switching in the non-autism group was also significantly and negatively correlated with lower Multidimensional Aptitude Battery scores, *r* = -0.51, *p* < .001; and lower Raven’s matrices scores. *r* = -0.28, *p* = .008.

### Re-meta-analysis

We next examined whether adding the current data to the meta-analysis of Zeif and Yechiam [13] would change its results. The analysis was run using the same exact approach as in the original paper, with a random effect model with generic inverse variance weighting and a restricted maximum-likelihood model estimator [39]. The results show that with the addition of the current dataset, the increased switching phenomenon is significant across studies, with Cohen’s *d* = 0.32, *Z* = 2.04, *p* = .04. Additional details of the re-meta-analysis are available in S3 File.

## Discussion

In an online sample of previously diagnosed autistic adults, we replicated the increased choice switching phenomenon formerly found in the lab [4, 9, 10]. Specifically, individuals in the autism group made on average 1.33 more switching decisions than in the non-autism group, with the difference being significant, consistently found in all trial blocks, and with the groups being compatible in gender, age, education, and general intellectual ability. When added to the results of the recent meta-analysis by Zeif and Yechiam [13], the choice switching phenomenon is significant across all studies, and the size of the effect is small to medium (*d* = 0.32).

In addition, the current findings shed some light on antecedents of the extreme choice switching phenomenon. First, the results are inconsistent with an explanation based on an implicit learning impairment: Learning rates were similar in both groups, and there was no significant correlation between increased choice switching and making fewer advantageous selections. Indeed, for the autism group the correlation between switching and advantageous selections was positive, though it did not reach significance. Secondly, the results are inconsistent with the notion that the effect is due to a sensitivity to losses (or any other feature of the feedback), as increased choice switching in autistic adults was also apparent in the trials with no feedback. Although this could be due to inertia, the finding of increased switching in the no feedback block in the autism group is incompatible with a simple model indicating that elevated switching occurs following losses. The absence of increased sensitivity to losses was also evident in our cognitive modeling analysis (expanded in the supplementary section).

Finally, the correlation between the switching rate and a diagnosis of OCD seems to indicate that at least part of the effect is due to a rigid and inflexible sampling strategy [10]. However, this analysis is based on smaller subsamples and indeed the model including both OCD and autism as independent factors showed a significant effect only for autism. This suggests that while autism was correlated with OCD, and autistic individuals with OCD showed the highest switching, autism trumped OCD as an explanatory factor for the group difference in switching. In addition, we did not find greater perseveration of the switching rate in different blocks in the autism group, which implies no increased rigidity in the strategy of option sampling. Further studies should evaluate the role of psychological rigidity (e.g., by using the Yale-Brown Obsessive Compulsive Scale [40, 41]) in the elevated switching phenomenon. Alternatively, the extreme switching of autistic individuals could be a form of exploration due to increased curiosity.

Importantly, increased sampling of the available options, either due to curiosity or rigidity, may underlie some of the phenomena previously attributed to poor learning in autism, such as slower category acquisition (see review in [16]), and even some of the social learning difficulties previously recorded in autism [8, 42]. Specifically, while in the current study elevated sampling was not correlated with poorer performance, this could be due to the fact that current task involves repeated trials. Given no or very few repetitions elevated sampling should in theory reduce performance. Because in this case both impaired learning and higher sampling can lead to similar learning impairments, it is important to somehow differentiate these two dimensions, by conducting experiments where elevated sampling results in clear predictions regarding performance level. One avenue for doing so is having multiple task repetitions which should eliminate the performance disadvantage of high sampling (see e.g., [43]), and in cases of inadequate initial solutions should even lead to a benefit for those using extensive sampling. A second avenue is by somehow identifying errors that are due to increased sampling compared to other types of errors, through reinforcement learning and other cognitive models (see supplementary section).

Reinforcement learning models applied to individual decision makers (or performers) can in theory disentangle the underlying cognitive effect of poor learning and increased sampling [4, 33]. However, it should be noted that state of the art models typically apply a stochastic choice rule, the so called ratio rule [44; and see S2 File], where increased sampling of choice option also implies lower sensitivity to the expected payoff structure, or in other words more random noise. However, as also suggested by the current findings, increased sampling may occur without reduced sensitivity to differences in payoff, which may require a refinement in the classical ratio rule. Indeed, learning models where sampling is a separate component are used in the decision-making literature (e.g., [45, 46]) but have not been applied to the analysis of individual differences as far as we know.

Another interesting and unexpected finding is that the SRS-2 scores in the non-autism group were rather high, with 35.1% of the participants scoring above the SRS-2 clinical threshold. There was also a correlation in this group between the SRS-2 scores and choice switching, with those reporting social difficulties getting to similar levels of switching as in the autism group (see S1 Fig). This guardedly suggests that social difficulties in study participants working for Mturk may exceed the norm in this questionnaire. This is consistent with the low social engagement and increased social isolation found in a recent study of Mturkers [47]. Nevertheless, group differences were found despite these elevated SRS-2 scores.

The most obvious limitation of the current findings is that it was not in the lab, and therefore we had little control about the diagnosis process. However, while this approach introduces a possible risk for the reliability of the diagnosis, this risk can be minimized by the addition of self-report questionnaires, which in the current study confirmed the difference between the two groups in autism-related symptoms. Another issue is the fact that there was no significant learning across trials in the non-autism group. However, this is a common outcome in IGT studies of general populations [48].

In future studies it would be interesting to examine how switching is related to overt knowledge acquisition in the task. For example, using a simplified version of the IGT, Faja et al. [49] found that autistic and non-autistic children exhibited a similar pattern of repeated choice selections, but autistic children evidenced less knowledge of the correct reward contingencies of the decks after playing. Besides testing the replicability of these findings for adults, it is worth examining whether the increased choice switching tendency, while maintaining an accurate implicit representation of the outcome, impairs the emergence of subsequent explicit knowledge, perhaps due to the difficulty of chunking the data.

To conclude, though making experiential choices with more frequent switches between options, autistic adults did not exhibit poorer learning, namely a tendency to select less advantageously, or showed greater loss sensitivity than a non-autism group. This along with the absence of a correlation between these factors suggests that elevated choice switching is not the outcome of poor learning or a motivation bias, but rather represents a distinct information sampling strategy that can perhaps account for other characteristics of autistic individuals’ behavior.

## Supporting information

S1 File Experimental instructions. (DOCX)

S2 File The Expectancy-Valence Prospect-Theory (EV-PT) model analysis. (DOCX)

S3 File Additional meta-analysis results. (DOCX)

S1 Dataset Study data. (XLSX)

S1 Fig Scatter plot of the relationship between choice switching and self-report tests. (DOCX)
