# Supplementary material for: Choice behavior in autistic adults: What drives the extreme switching phenomenon?
Source: PLoS One. 2023 Mar 2;18(3):e0282296. doi: 10.1371/journal.pone.0282296 (PMC9980774; doi:10.1371/journal.pone.0282296)

Experimental instructions:

Initial survey (session 1)

Enter your age _____

What gender do you identify as? 🔾 Male, 🔾 Female, 🔾 Other

Were you ever diagnosed with any brain injury of some kind? 🔾 Yes, 🔾 No

If you were ever diagnosed with any neurological disorders, please specify_____

Level of highest education obtained 🔾 Pre School, 🔾 High School, 🔾 College,

🔾 Master's or Professional Degree, 🔾 PhD

Do you have a formal diagnosis of OCD? 🔾 No, 🔾 Yes

Do you have a formal diagnosis of Schizophrenia? 🔾 No, 🔾 Yes

Diagnosis File Upload Instructions
In order to complete the experiment, we will have to verify that you have a formal autism spectrum disorder diagnosis.
Your privacy is important to us and we will delete these files from the secure server shortly.
**In order to keep your privacy and anonymity- please remove any personal information from the file you are uploading (ID, name, etc.)**
Please upload a formal document signed by a licensed professional, which confirms you were diagnosed with autist spectrum disorder. The document (pdf, image, etc.) should be readable.

Iowa Gambling Task Instructions

In front of you, there are four decks of cards labelled A, B, C, and D.

Your task is to select one card at a time from any deck you choose.

Each time you select a card you will get some money. You will find out how much as the task progresses. Every so often, however, you will lose some money too. You will find out how much as the task progresses.

You are absolutely free to switch from one deck to the other at any time, and as often as you wish.

The goal of the game is to win as much money as possible, or avoid losing money as much as possible.

You won't know when the game will end. You must keep on playing until you get a message to stop.

It is important to know that the colors of the cards are irrelevant in this game and that there is no way for you to know in advance what the payoffs will be. All we can say is that some decks are worse than others. No matter how much you find yourself losing, you can still win if you stay away from the worst decks. Please treat the game money in this game as real money, and any decision on what to do with it should be made as if you were using your own money.

You’ll now get a loan of $3500 of game money.

At the end, we will collect back the loan and see how much you won or lost such that every $1500 of game money will be converted to $1 real money.

No Feedback Block Instructions

Note that from this point you will not receive feedback on your choices.

IGT screen when making a selection:


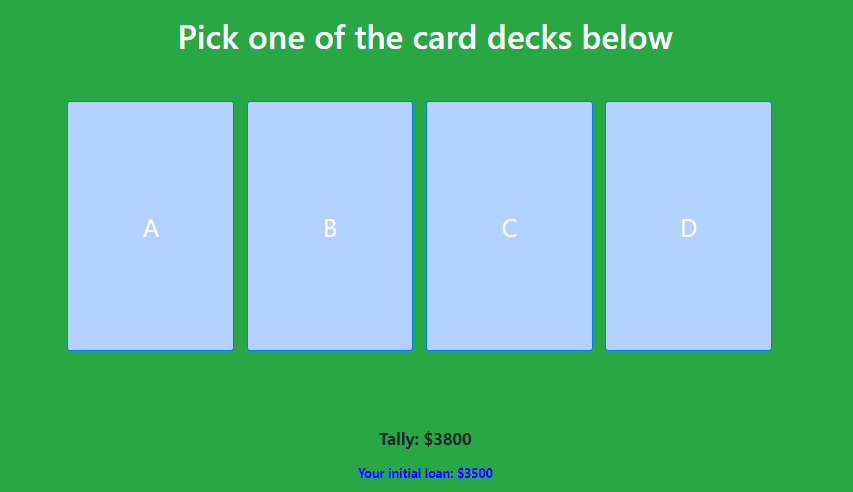


IGT screen following a selection:


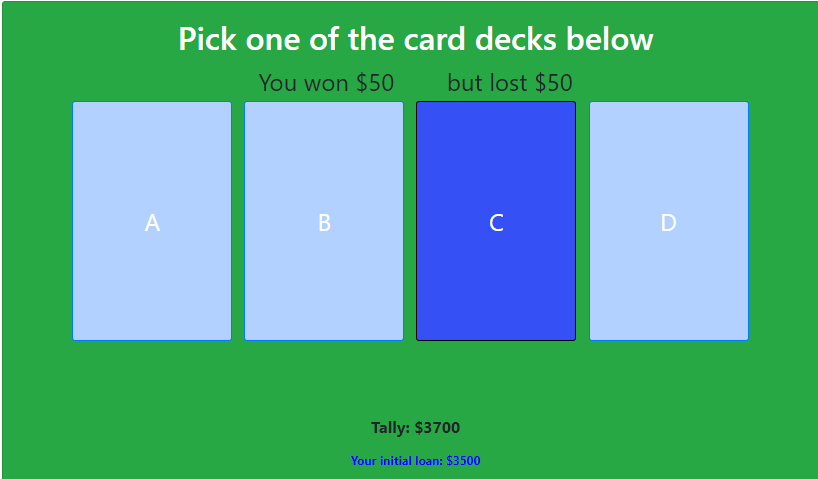

Supplement: S1 File — (DOCX) [file pone.0282296.s001.docx]
