# Supplementary material for: Choice behavior in autistic adults: What drives the extreme switching phenomenon?
Source: PLoS One. 2023 Mar 2;18(3):e0282296. doi: 10.1371/journal.pone.0282296 (PMC9980774; doi:10.1371/journal.pone.0282296)
Supplement: S2 File — (DOCX) [file pone.0282296.s002.docx]

The Expectancy-Valence Prospect-Theory (EV-PT) model analysis:

The EV-PT model [35] is a computational model analyzing trial-by-trial choice-behavior. It distills the performance in the IGT (and similar tasks) into three latent components providing a cognitive-motivational profile of each individual decision-maker.

1) Loss aversion: The increased/decreased sensitivity to losses compared to gains is calculated using a formula similar to that of prospect theory [S1], taking into account the gains and losses from the chosen option in trial *t*.

| *u*(*t*) = *win*(*t*) − *λ*⋅*loss*(*t*) , | (1) |
| --- | --- |

where *u*(*t*) is the utility of the outcome, and the parameter *λ* determines the degree of loss aversion: how much an individual give more weight to losses than gains, or vice versa (0 ≤ *λ* ≤ 10).

2) Recency: The utilities produced by deck *j* are summarized by an accumulated subjective value for each deck, called an expectancy, and denoted *Ej*(*t*). A decay-learning rule updates the expectancy after each choice from deck *j*:

| *E_j_*(*t*) = *φE_j_*(*t*-1) + *u*(*t*) , | (2) |
| --- | --- |

The recency parameter, *φ*, describes the degree to which past expectancies are decayed compared to new experience (0 ≤ *φ*  ≤ 1).

3) Choice consistency. The predicted probability that deck *j* will be selected on trial *t*, Pr[*Gj*(*t*)], is calculated using a ratio of strengths rule [44]:

| $\Pr[G_{j}(t)]=\frac{e^{\theta(t)\cdot E_{j}(t)}}{\sum_{j} e^{\theta(t)\cdot E_{j}(t)}}$ , | (3) |
| --- | --- |

The term *θ* (*t*) modulates the consistency of choice probabilities and expectancies, where: *θ* (*t*) = (*t*/10) *^c^* and *c* is the choice consistency parameter. Notice that low choice consistency implies more sampling of choice options and greater choice switching. The parameter *c* is constrained between 5 and -5, covering the range between deterministic expectancy-based choices and nearly random choices, respectively.

Estimation and baseline models: The parameters of the Expectancy-Valence (EV) model were optimized separately for each participant by maximizing the likelihood of choices in trial t+1 given the experiences in the previous trials and the model parameters [see details in 4,35]. The fit of the EV-PT model was compared with the fit of a baseline model, which does not assume any learning, and has three parameters based on the average choice proportions of Decks A, B, and C.

The improvement in the fit of the EV-PT model compared the baseline model, in log likelihood, is *G*^2^, a model-fit statistic analogous to the chi-square. Positive *G*^2^ values indicate that the model outperforms the baseline model, whereas negative values indicate the opposite. In addition, though the EV-PT was found to outperform the delta-learning model [S2] used in Johnson et al. [4], we also examined the prediction of the latter model for robustness, and also to replicate the exact formulation used in [4]. Notice that all of these three models (EV-PT, delta-learning, baseline) have the same number of parameters (three) requiring no model fit corrections. Because the models have three parameters, all group comparisons used appropriate Bonferroni corrections for three examinations.

Results:

The average *G*^2^ of the EV-PT model for the autism group was 22.58 ± 6.69 and for the non-autism group it was 39.56 ± 6.49. Thus, in both groups the learning model better fit the data than the baseline model (which does not assume any learning).

The estimated EV-PT model parameters appear in Table S1 below. The only significant difference between groups was for the choice consistency parameter (*t*(112) = 2.69, *p* = .01), with lower choice consistency in the autism group. Differences were not significant for loss aversion and recency. For the delta model [4], model fits were lower compared to the baseline model (autism group *G*^2^ = -5.28 ± 2.40, non-autism group *G*^2^ = 2.01 ± 4.90), and there were no significant differences in any of the parameters (see Table S1).

Table S1. Estimated parameters for the EV-PT and delta models. Means and standard deviations (in parentheses) in the autism and non-autism group.

|  | EV-PT | | Delta | | |
| --- | --- | --- | --- | --- | --- |
|  | Autism | Non-Autism | | Autism | Non-Autism |
| Loss aversion (*λ*) | 1.39 (0.41) | 0.75 (0.31) | | 0.39 (0.29) | 0.39 (0.29) |
| Recency (*φ*) | 0.38 (0.04) | 0.48 (0.04) | | 0.38 (0.04) | 0.27 (0.03) |
| Choice consistency (*c*) | -0.05 (0.06)* | 0.31 (0.12)* | | -0.92 (0.20) | -0.44 (0.16) |

Notes: * = p < .05 in the comparison of the autism group to the non-autism group, using Bonferroni corrected t-tests (p-values were multiplied by three).

References

S1. Kahneman D, Tversky A. Prospect theory: An analysis of decision under risk. Econometrica. 1979; 47:263-291.

S2. Busemeyer JR, Stout JC. A contribution of cognitive decision models to clinical assessment: Decomposing performance on the Bechara gambling task. Psychological Assessment. 2002; 14:253-262.
