# Supplementary material for: Choice behavior in autistic adults: What drives the extreme switching phenomenon?
Source: PLoS One. 2023 Mar 2;18(3):e0282296. doi: 10.1371/journal.pone.0282296 (PMC9980774; doi:10.1371/journal.pone.0282296)

Additional meta-analysis results:

Supplementary Figure 3: Forest plot for choice switching differences between the autism and non-autism groups in a re-meta-analysis of Zeif and Yechiam (2020) with the addition of the current study. Effect sizes falling to the left of zero indicate more switching for the autism group. Error bars represent 95% confidence intervals (CI) around the effect sizes. The right hand side presents the effect sizes and CI in brackets for each study. Below is the random effect (RE) model estimate.


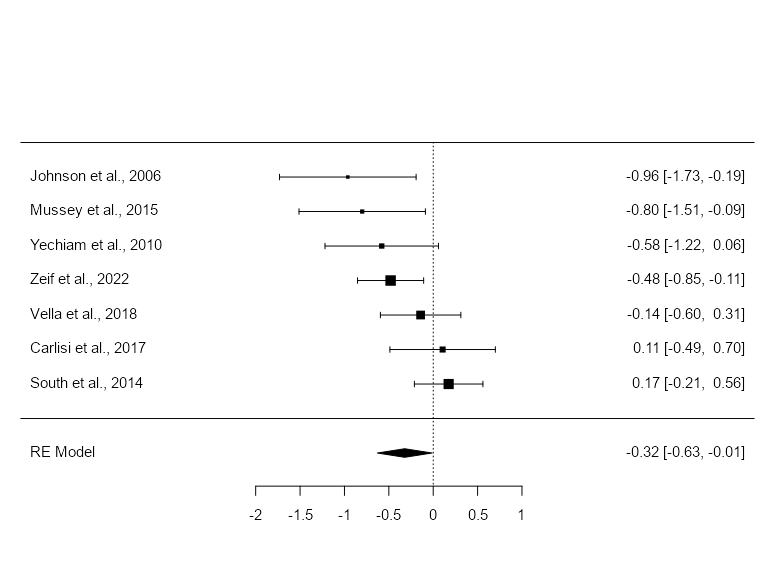


Supplementary Figure 4: Funnel plots displaying the inverse standard error of the effect size (Cohen’s *d*), plotted as a function of the magnitude of the effect size in each study. The dotted lines denote the predicted 95% confidence intervals. Although a slight visual asymmetry is apparent, no significant publication bias was detected in Egger’s regression (*Z* = -1.64; *p* = .19).


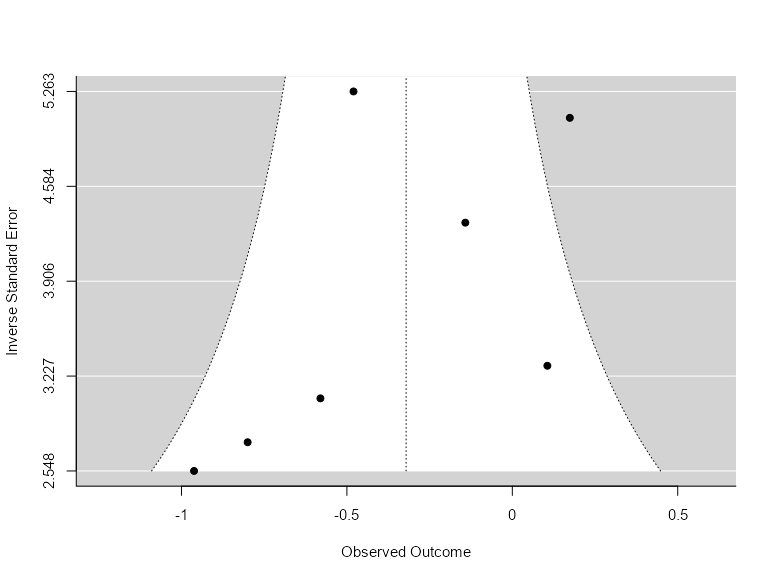

Supplement: S3 File — (DOCX) [file pone.0282296.s003.docx]
