# Supplementary material for: Choice behavior in autistic adults: What drives the extreme switching phenomenon?
Source: PLoS One. 2023 Mar 2;18(3):e0282296. doi: 10.1371/journal.pone.0282296 (PMC9980774; doi:10.1371/journal.pone.0282296)

S1 Fig: Scatter plot of the relationship between choice switching and self-report tests. The relationship between choice switching and Social Responsiveness Scale (SRS-2 scores) (left panels; blue), and Autism Quota (AQ scores) (right panels; orange), in the autism and non-autism groups. Trendlines denote the (predicted) results of a linear regression.

**Autism Group**


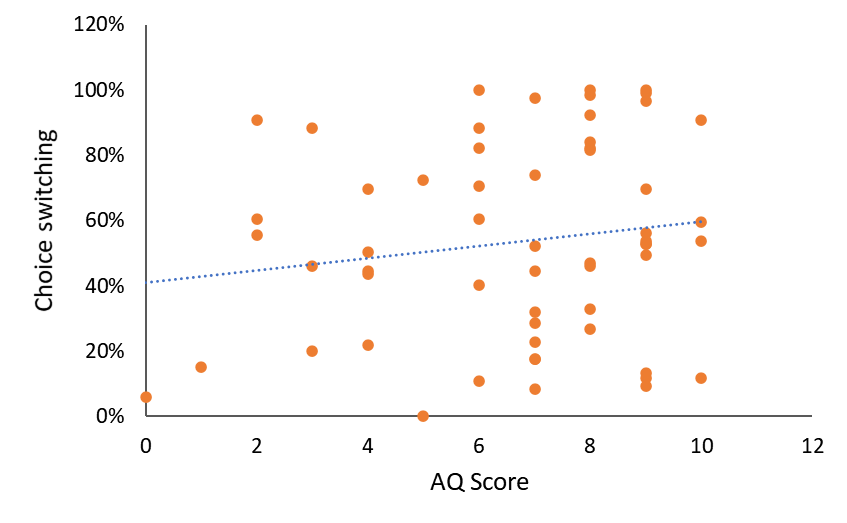

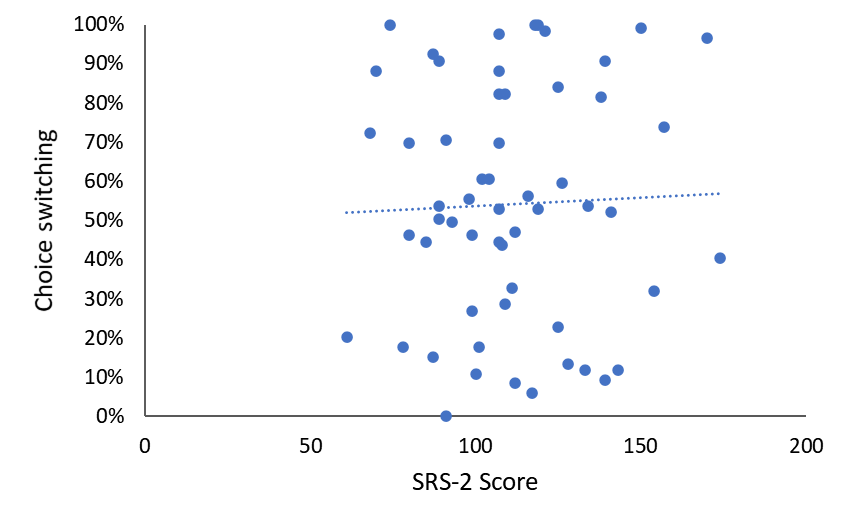

**Non-Autism Group**


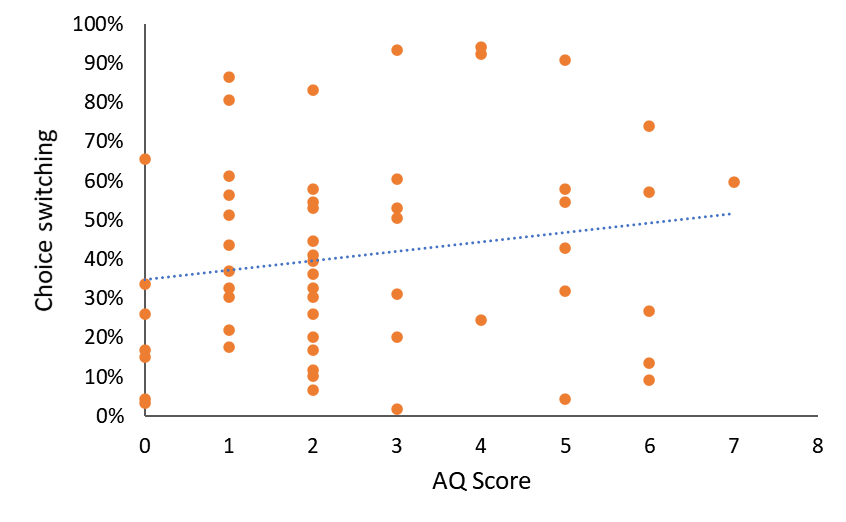

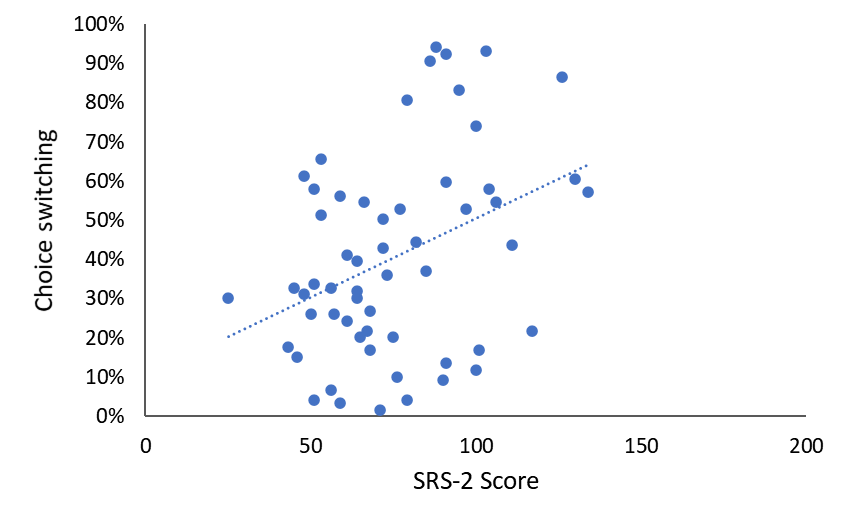

Supplement: S1 Fig — (DOCX) [file pone.0282296.s005.docx]
